# A 3D Co-Culture Scaffold Approach to Assess Spatially Fractionated Radiotherapy Bystander and Abscopal Immune Effects on Clonogenic Survival

**DOI:** 10.3390/ijms26094436

**Published:** 2025-05-07

**Authors:** Nicholas Casteloes, Carrie D. House, Mauro Tambasco

**Affiliations:** 1 Department of Physics, San Diego State University, San Diego, CA 92182, USA; ncasteloes8869@sdsu.edu; 2 Biology Department, San Diego State University, San Diego, CA 92182, USA; cdhouse@sdsu.edu

**Keywords:** spatially fractionated radiotherapy (SFRT), 3D cell culture, co-culture, bystander effect, abscopal effect, tumor microenvironment, breast cancer, PBMCs, clonogenic survival, immune response, GRID-RT

## Abstract

Spatially fractionated radiotherapy (SFRT) offers a promising approach for debulking large tumors by delivering high-dose radiation to a fraction of the tumor volume. However, the complex tumor microenvironment necessitates models beyond traditional 2D cultures and resource-intensive animal studies for SFRT investigations. Three-dimensional (3D) scaffold-based models with an adequate cross-sectional area have emerged as uniquely suited platforms to bridge this gap, by providing a more realistic platform for GRID-based SFRT research. In this study, we employed a 3D co-culture scaffold model to dissect the contributions of the radiation-induced bystander effect, abscopal effect, and immune system response on clonogenic survival following GRID irradiation. MDA-MB-231 breast cancer cells were seeded on commercial 3D scaffolds and irradiated at a 20 Gy peak dose using lead grids with three- and six-hole patterns, exposing ~12.8% and 25.7% of the scaffold area, respectively. An assessment of reproductive cell survival revealed a significant bystander effect, as the survival was notably lower than predicted based solely on the directly irradiated fraction. Evidence of an abscopal effect was observed by culturing non-irradiated cells in media exposed to GRID irradiation. Furthermore, a co-culture with allogeneic peripheral blood mononuclear cells (PBMCs) modulated clonogenic survival, with an additive effect observed when combined with SFRT. These findings underscore the presence of a bystander effect in GRID radiotherapy and indicate an abscopal immune component, particularly with the three-hole GRID configuration. This study established the utility of in vitro 3D co-culture scaffolds as an effective model system for elucidating complex SFRT-mediated biological responses.

## 1. Introduction

Conventional radiotherapy can damage surrounding tissues, leading to mild, moderate, or severe short-/long-term morbidity of the damaged tissue. Spatially fractionated radiotherapy (SFRT) is clinically used to debulk large tumors, by using a large non-uniform dose of 10–20 Gy, irradiating up to 50% of the volume to the maximum dose [1,2,3,4]. SFRT is followed by conventional fractionation to complete the treatment. This approach has been shown to significantly reduce normal tissue toxicity [3].

Early radiotherapy techniques that used superficial/orthovoltage energies were effective at reducing normal tissue damage using GRID-based SFRT, but this approach diminished with the widespread adoption of powerful skin-sparing megavoltage (MV) X-ray treatments [4]. Currently, GRID and LATTICE radiotherapy (RT) are the two main forms of SFRT used to debulk large tumors. GRID-RT uses either a physical collimator constructed from a high z material or Linac’s multi-leaf collimators to limit the primary beam to small regions in either a hexagonal or orthogonal pattern to create a distribution of peak and valley doses. For physical GRID collimators, the peak doses range from 10 to 20 Gy, while the valleys typically receive 15–20% of the primary dose for a 6 MV beam, and approximately 30% for an 18 MV beam [2]. In contrast, LATTICE radiotherapy typically uses volumetric modulated arc therapy or proton radiotherapy to create peak-dose spheroids comprising 1–10% of the gross tumor volume and yielding a peak-to-valley dose ratio ranging from 2 to 5% [5,6].

In addition to reducing normal tissue toxicity, in vitro SFRT techniques have been shown to elicit the radiation-induced bystander effect (RIBE) [3,7]. In 1992, the bystander effect, a local response, was first observed [8]. This effect, primarily mediated by communication between cells and exposure to cytotoxic materials, occurs on a small scale and results in the death of non-irradiated cancer cells [9]. This effect contrasts sharply with the conventional approach of irradiating the entire tumor in many small dose fractions to kill the cancer cells while minimizing damage to surrounding normal cells. By employing SFRT, this bystander effect can be harnessed to yield greater therapeutic advantages, such as less normal tissue damage for the same tumor control compared to conventional treatments because a significantly lower normal tissue volume is irradiated [3].

SFRT alone has also been shown to occasionally induce an abscopal effect. In contrast to the local nature of the bystander effect, which occurs only in the primary tumor, the abscopal effect is defined as the regression of distant metastases following primary tumor irradiation. The principal mechanism for the abscopal effect is thought to stem from the immune system being stimulated by the irradiation of the primary tumor [10].

To investigate the magnitude and mechanisms of action of the GRID-RT bystander and abscopal effects in vitro, two-dimensional (2D) cell cultures, 3D spheroids, or preclinical murine models have been used [7,11,12,13]. While 2D adherent cell cultures have been the standard in vitro model for decades in radiotherapy research, they have been shown to inadequately replicate in vivo conditions and typically only allow for the study of a single cell type [14,15]. Hence, to better replicate in vivo conditions of human tumors, researchers have increasingly shifted from 2D cell culture studies to 3D cell cultures [15,16]. Three-dimensional cultures exhibit enhanced radio-resistance compared to their 2D adherent counterparts, fostering a more in vivo-mimicking environment [17,18]. Spheroids are the most common 3D model in radiobiology, but their size (≤750 µm) limits their use in studying SFRT even with mini-beam radiotherapy, which utilizes SFRT beamlets of 500–700 µm [19,20,21]. Although synchrotron microbeam radiation therapy (MRT) provides a more suitable beamlet scale of 25 to 100 µm for GRID-RT spheroid studies, MRT is not used clinically, and its availability is very limited [22]. Additionally, while attempts have been made to study the bystander effect using 3D cultures and by employing alpha particle microbeams or radiolabeled nucleotides, these approaches are not clinically available [23].

Murine models, though valuable for studying immune responses to murine cancers, are a little more challenging to irradiate, and they have inherent differences in their biological response to radiation and other therapies that can limit their clinical translation [24,25]. More importantly, they are also significantly more expensive than in vitro models and necessitate careful consideration of animal ethics. Hence, to bridge the gap between existing in vitro and murine studies, and to enable GRID-RT research with standard X-ray equipment, alternative models that reduce animal reliance are essential.

In this study, we explored the utility of commercially available 3D cell culture Alvetex^®^ scaffolds (REPROCELL USA Inc., Beltsville, MD, USA) to investigate the bystander and abscopal effects of GRID-RT. Such scaffolds have found applications in cancer research, primarily in the context of anti-cancer drug development, but they have been underutilized in radiotherapy research [26,27,28]. A significant benefit of using 3D scaffolds for GRID-RT studies is their well-defined, relatively large, flat cross-sectional areas ranging in diameter from 11.5 to 22 mm. Hence, while numerous 3D culture systems exist for spheroid and organoid models, the 3D porous polystyrene substrate Alvetex^®^ scaffolds offer a convenient platform for generating small spheroids, which are known to exhibit a morphology and cell–cell communication that are more representative of in vivo tumor conditions than traditional 2D cultures [29].

To our knowledge, there have been limited applications of 3D scaffolds in radiobiology studies [15]. Additionally, previous SFRT studies have not explored the abscopal effect using in vitro 3D co-cultures of cancer cells and peripheral blood mononuclear cells (PBMCs). To address this gap, we developed an in-house 2D collimation grid with dosimetry validation to deliver GRID-RT to cancer cells grown in a 3D co-culture scaffold. As proof of concept, we used MDA-MB-231, a highly aggressive and metastatic breast cancer cell line, and the second most frequently used breast cancer cell line in cancer biology and immunology research [30]. We introduced allogeneic PBMCs to the 3D breast cancer culture post-irradiation to investigate a possible immunomodulatory response induced by the GRID treatment that is above and beyond the allogeneic response. This allogeneic co-culture combination has been previously utilized in 3D cancer studies and has demonstrated the ability to reduce MDA-MB-231 viability [31]. The monocytes and T cells present in the PBMCs are required for the observation of the long-range bystander and abscopal effects, respectively [32].

Additionally, we studied the impact of GRID irradiation on these effects by systematically varying parameters, including the fraction of irradiated area, the presence of PBMCs, and the time allowed for cell repopulation. We assessed reproductive cell survival using the clonogenic assay, the gold standard for this endpoint in radiobiology studies. The significance of this study lies in its exploration of bystander and abscopal effects resulting from GRID irradiation using a 3D co-culture scaffold model. This study paves the way for future studies investigating the effects of parameters such as the GRID peak dose, valley dose, peak-to-valley dose ratio, percent irradiated volume for standalone GRID irradiation, and immune system modulation. By establishing a robust model capable of analyzing these effects, the role of SFRT (i.e., GRID, LATTICE, and other related approaches) as potentially potent immunomodulators can be accelerated, ultimately leading to improved tumor control and reduced normal tissue complications.

## 2. Results

### 2.1. Dosimetry

**Figure 1:**
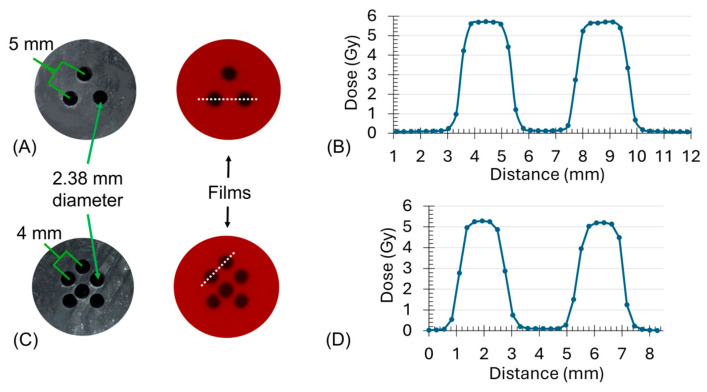
The construction and dosimetry of lead grid collimators for GRID irradiation. (**A**,**B**) show the 3- and 6-hole grid patterns and their corresponding Gafchromic^TM^ EBT3 films, respectively, irradiated to 5 Gy. Note: Dotted lines in the films shown in (**A**,**C**) indicate the corresponding profiles plotted in (**B**,**D**), respectively. (**C**,**D**) show the representative profile dose measurements for the 3- and 6-hole grids, respectively.

Using the film calibration curve, we found the beam flatness to be 4.2 ± 0.4%. Additionally, irradiating our film within the 24-well plate with the attached lead grid resulted in a median dose of 0.40 ± 0.12 Gy to the shielded areas, with doses ranging from 0.28 to 0.54 Gy (Figure 1). With the irradiation time set to deliver 5 Gy, areas within the primary beam produced the expected results. The average dose of the primary beamlet locations was 5.3 Gy, falling within the calibration curve uncertainty of 9% (5.0 ± 0.4 Gy).

### 2.2. Clonogenic Survival for All Cell Culture Groups

The MDA-MB-231 clonogenic survival for all the study groups is summarized in Table 1.

### 2.3. Local Effect of PBMCs Alone (C2 vs. C1)

**Figure 2:**
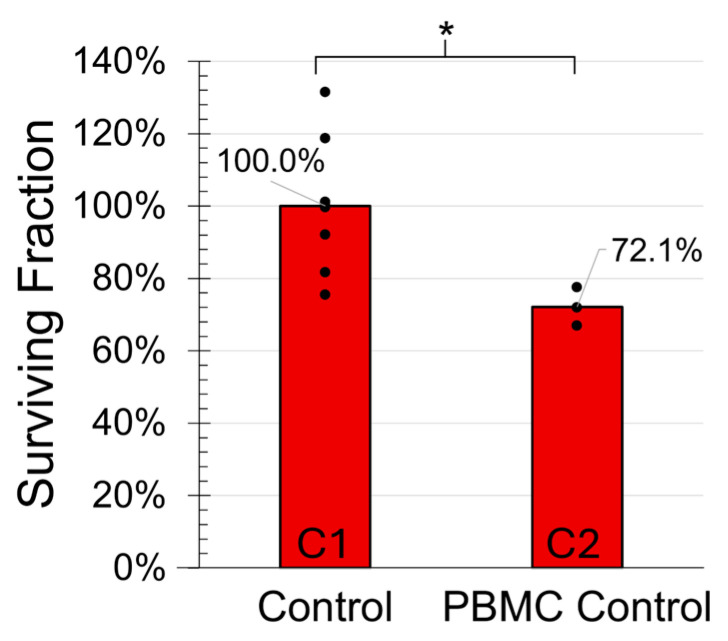
Clonogenic survival of MDA-MB-231 cells for the control and PBMC control groups demonstrated an allogeneic immune response. Data points indicate biological replicates and * signifies *p* < 0.05.

The reproductive cell survival between the PBMC control (C2) was significantly less than the control (C1) (*p* < 0.05; Figure 2), demonstrating an allogeneic immune response.

### 2.4. Effect of Irradiated Conditioned Media (ICM) Alone (1A/1B vs. C1)

**Figure 3:**
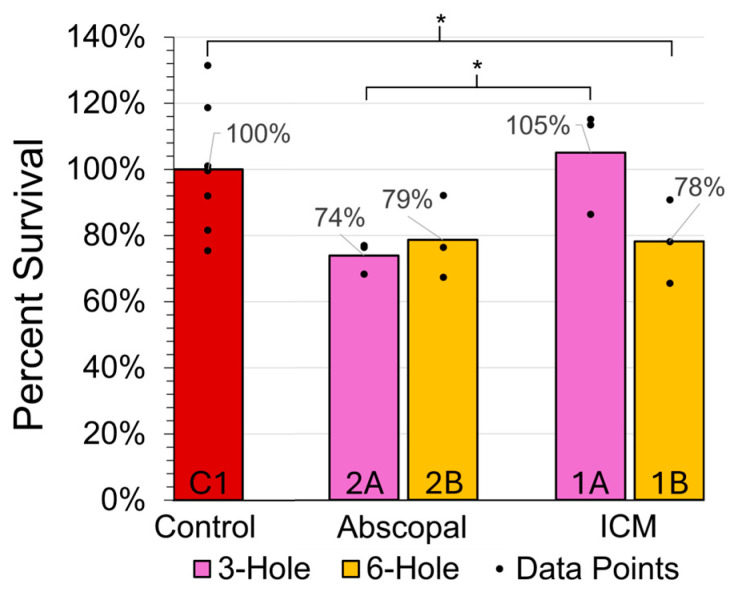
The effect of ICM on MDA-MB-231 clonogenic survival in the 3D scaffolds with the addition of PBMCs (abscopal groups 2A and 2B) and without PBMCs (ICM groups 1A and 1B) for both the 3- and 6-hole GRID irradiations. The data points indicate biological replicates and * signifies *p* < 0.05.

Non-irradiated MDA-MB-231 cells incubated in ICM derived from three-hole GRID irradiation showed no statistically significant differences in the reproductive cell survival if they were given sufficient time to repair and repopulate. In contrast, non-irradiated MDA-MB-231 cells in ICM derived from six-hole irradiation showed a significant decrease, even after 5 days of repair and repopulation (Figure 3). Assuming the six-hole GRID irradiation group induced twice as many bystander-effect signaling molecules than the three-hole group, this suggests that the decreased reproductive cell viability due to these molecules is concentration-dependent.

### 2.5. Test for Abscopal Effect (2A/2B vs. 1A/1B, Respectively)

The addition of PBMCs to non-irradiated MDA-MB-231 cells (i.e., group 2A, representing metastasis) led to a statistically significant decrease in the reproductive survival versus group 1A, without PBMCs (*p* < 0.05), suggesting the presence of an allogeneic abscopal effect (Figure 3, Table 1). However, as shown in Figure 3, no differences were found between the six-hole groups (i.e., 2B vs. 1B). Also of note is that the survival rates of the three-hole and six-hole abscopal groups (2A vs. 2B) did not differ significantly from each other (Figure 3).

### 2.6. Radiation Alone to Test for Bystander Effect (3A/3B vs. Expected Survival)

**Figure 4:**
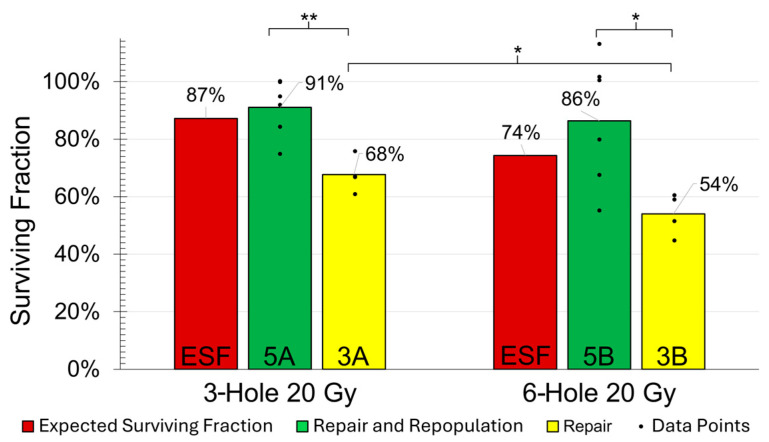
Comparison of MDA-MB-231 clonogenic survival after 20 Gy 3-hole and 6-hole GRID irradiations, allowing for either 1 day of repair (3A and 3B) or 1 day of repair followed by 5 days of repopulation (5A and 5B). The expected surviving fraction (ESF) represents the minimum percentage of cells expected to survive, based solely on the proportion of the area that was not exposed to radiation. It is a lower-bound estimate of cell survival, derived from the non-irradiated area (see Section 4.13 for details). The data points indicate biological replicates; * and ** indicate statistical significance with *p* < 0.05 and *p* < 0.01, respectively.

The mean percent reproductive survival was 19% and 20% lower for GRID irradiations of 3D scaffolds using the three- and six-hole grids compared to the expected survivals of 87.2% and 74.3%, respectively (Figure 4). This difference indicates the presence of a bystander effect. However, the reduced clonogenic capacity was not directly proportional to the sum of the hole perimeters (see Section 3). It is also worth noting that, after a 24 h repair period, the mean reproductive survival of the 3D scaffold cultures irradiated to a 20 Gy peak dose, group 3A, was notably greater (*p* < 0.05) than that of group 3B, as expected (Figure 4).

### 2.7. Effect of Repopulation (5A/5B vs. 3A/3B, Respectively)

As expected, allowing for 5 days of repopulation after 1 day of repair from GRID irradiation resulted in a significant increase in the reproductive survival for both the three- and six-hole GRID groups, with *p* < 0.01 and *p* < 0.05, respectively (Figure 4).

### 2.8. Local Effect of Radiation + PBMCs vs. Radiation Alone (4A/4B vs. 5A/5B, Respectively)

**Figure 5:**
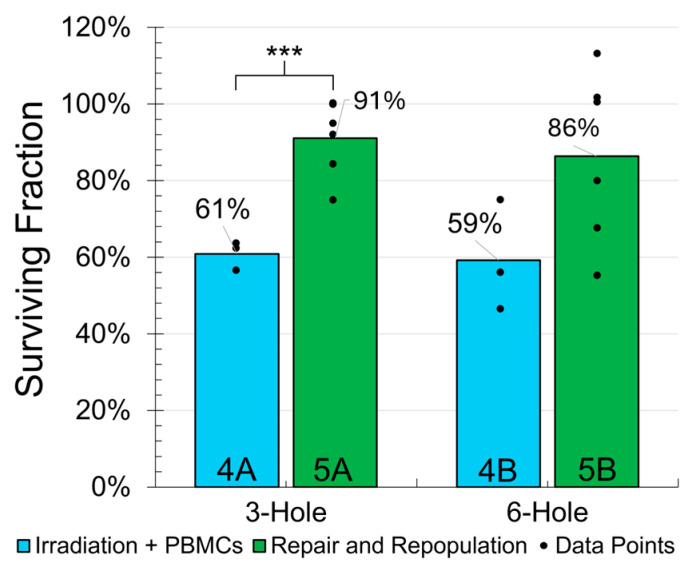
Comparison of clonogenic survival for GRID irradiation + PBMCs (4A and 4B) versus GRID irradiation alone (5A and 5B), both with repair and repopulation. The data points indicate biological replicates; *** indicates statistical significance with *p* < 0.001.

While the PBMC addition post-irradiation significantly reduced the MDA-MB-231 cell reproductive survival in the three-hole GRID group (*p* < 0.001), a substantial 27% difference in the mean survival for the six-hole GRID group did not achieve significance due to the large data deviation within the 5B group (cf., Figure 5 and Table 1).

## 3. Discussion

To our knowledge, this study is the first to use 3D cell culture scaffolds with and without immune cells to study the bystander and abscopal immune system effects of GRID irradiation. For basic and preclinical GRID radiotherapy research, 3D scaffold models offer a significant advancement over 2D cell cultures and murine models, providing a balance of increased complexity, cost-effectiveness, and high-throughput capabilities for the efficient investigation and optimization of radiotherapy before animal trials. The primary objective of this study was to determine if 3D scaffolds, with their substantial cross-sectional area, could offer an effective 3D co-culture platform for studying bystander and abscopal immune response effects of GRID irradiation on clonogenic cell survival. The findings presented here demonstrate the utility of this approach and provide insights for guiding future experiments by utilizing 3D scaffolds for radiation therapy research, as well as informing future pre-clinical murine model research.

### 3.1. Observation of the Radiation-Induced Bystander Effect

While a single in vitro study quantified the RIBE from spatially varying radiation fields using two types of normal 3D-reconstructed human skin tissue [33], the majority of in vitro studies have primarily focused on 2D adherent cultures [11,12,13,34]. Notably, the study involving 3D normal tissue utilized microbeam α-particles with a diameter of less than 5 μm and observed the bystander effect up to 1 mm away from cells irradiated with a low 1 Gy dose [33].

All the aforementioned 2D studies used one or more cell lines and clonogenic survival as the endpoint, and they reported the presence of the RIBE in collimated regions. In a separate study, Maeda et al. irradiated V79 cells with synchrotron X-ray microbeams and observed that the RIBE extended at least out to 3 mm following a whole-cell radiation dose of 2.1 Gy [35]. Some of the 2D studies used large single-fraction doses of at least 8 Gy and as much as 30 Gy; however, they did not explicitly measure the spatial extent of the RIBE [36,37,38].

Like the majority of these 2D studies, we did not explicitly measure the spatial extent of the RIBE. Our isolation of this effect on reproductive cell survival from 20 Gy peak-dose GRID irradiation demonstrated a 19% and 20% lower reproductive survival than anticipated for both the three- and six-hole samples, respectively (Figure 4). This similar decrease for both groups suggests that the relationship between the irradiated GRID regions and the bystander effect are not linear. That is, doubling the total area and the corresponding total perimeter distance of the grid holes did not appear to lead to a proportional increase in the RIBE.

This non-linear relationship may be attributed to a high-dose GRID bystander effect occurring over a distance of at least 1.6 mm, leading to an “overkill” effect in the overlapping bystander regions between the holes. As the number of irradiated regions increases, the overlapping regions between primary irradiated zones also increases. This overlap could lead to the excessive accumulation of bystander signals in these regions, resulting in the saturation or even a decline in the bystander effect beyond a certain threshold of total irradiated area. These results highlight the importance of carefully considering the spatial distribution of the radiation dose when designing SFRT treatments to optimize the therapeutic benefit while minimizing the potential negative consequences of the bystander effect.

In agreement with our study, several investigations using spatially varying radiation fields and 2D monolayer cell cultures have observed the RIBE in the collimated valley regions [13,36,38,39]. Collectively, our study and these previous studies highlight the crucial role that grid spacing and the total volume irradiated all play in eliciting the RIBE. However, the specific contributions of these parameters will need to be further assessed in future studies by varying the shielding transmission, spacing patterns, hole sizes, and total number of holes.

### 3.2. Observation of an Immune System Response

Our results indicate that the baseline allogeneic immune response alone played a role in reducing the survival of MDA-MB-231 cancer cells in our 3D scaffold co-culture model. We observed a 30% reduction in cell viability when PBMCs were present, which is consistent with a previous study using MDA-MB-231 3D spheroids and PBMCs without chemical immune system priming [31]. Radiation with subsequent PBMC co-cultures further decreased the survival, but this reduction was additive rather than synergistic (Figure 5 and Table 1). This suggests that the radiation dose did not induce an immune response that went above and beyond the allogeneic immune effect. A possible reason for this could be that radiation exposure is also known to increase the expression of programmed death ligand 1 (PD-L1) in MDA-MB-231 cells, which would have inhibited T cell function by binding to programmed cell death protein 1 (PD-1) on T cells, thereby dampening any radiation-induced immune responses [40,41].

Our experiments, which simulated the abscopal effect by transferring non-irradiated cells to ICM, media that bathed irradiated cells, support this finding. GRID-treatment-derived ICM alone did not trigger a significant immune response, as irradiated cell contents combined with PBMCs yielded survival rates similar to PBMCs alone (Table 1: C2 vs. 2A and 2B). These results suggest that the primary driver of cell death for MDA-MB-231 is the direct allogeneic effect of the PBMCs.

### 3.3. Study Limitations

Our study has some limitations that warrant consideration. Firstly, we used a single breast cancer cell line, MDA-MB-231. While this approach has also been taken by other researchers and allows for a focused analysis [35,36], it does limit the generalizability of our results to other subtypes of breast cancer or different cancer types. Future studies could investigate the bystander and abscopal effects in other cancer cell lines or patient-derived tumor cells to better understand the potential variability in these responses. Secondly, while we observed a high-dose bystander effect in MDA-MB-231 cells, an investigation of the underlying biological mechanisms responsible for this effect was beyond the scope of this study. Further research is needed to elucidate the specific pathways involved in this phenomenon. Thirdly, although the use of allogeneic PBMCs has value in immunomodulation cancer studies [31], it does not fully recapitulate the more clinically relevant autologous immune response. Lastly, while the 3D scaffolds employed in this study effectively model the bystander effect and immune system responses, they cannot fully recapitulate the vascular and stromal effects observed in tumors.

### 3.4. Future Studies

Future studies will incorporate autologous PBMCs and will also include immune checkpoint inhibitors (e.g., PD-1/PDL-1) to assess the role of these immunotherapies for increasing the immunomodulatory effects of radiation.

## 4. Materials and Methods

### 4.1. Experimental Workflow and Cell Culture Group Comparisons

**Figure 6:**
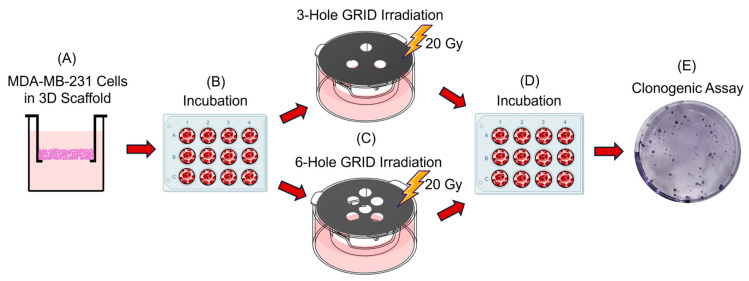
Experimental workflow. (**A**) MDA-MB-231 cells were seeded onto 3D scaffolds. (**B**) The cells were incubated in a 12 well plate for 5 days (Created in BioRender. Casteloes, N. (2025) https://BioRender.com/zt3bc5s, accessed on 4 May 2025) (**C**) The 3D scaffold cell cultures were irradiated with 3- or 6- hole GRID. (**D**) Cells in the scaffolds were either allowed to repair for 1 day and removed from the incubator for processing or kept in the incubator for an additional 5 days of repopulation. (**E**) The cells were extracted from the scaffolds and plated to assess the reproductive cell survival.

Figure 6 shows the experimental workflow used in this study to assess the RIBE and abscopal effects of GRID irradiation on the MDA-MB-231 metastatic breast cancer cell line.

All irradiations were delivered in a peak dose of 20 Gy using our in-house SFRT 3- and 6-hole grid patterns described below. Using the 3D culture scaffold group definitions given in Table 1, the experimental design included the following six distinct comparisons in biological triplicate:

1. Local effect of PBMCs alone (C2 vs. C1): Non-irradiated 3D MDA-MB-231 cell scaffolds were co-cultured with PBMCs (1:1 ratio) for 5 days and compared with non-irradiated controls.
2. Effect of irradiated conditioned media (ICM) alone (1A/1B vs. C1): Non-irradiated MDA-MB-231 cultures were incubated for 5 days in ICM derived from either 3- or 6-hole GRID irradiations. This group served as a control for the abscopal effect.
3. Test for abscopal effect (2A/2B vs. 1A/1B, respectively): Non-irradiated MDA-MB-231 culture scaffolds (representing metastases) were co-cultured with PBMCs (1:1 ratio) for 5 days in ICM derived from the 3- or 6-hole GRID irradiation of MDA-MB-231 scaffolds. These were compared to MDA-MB-231 cells cultured in ICM without PBMCs.
4. Radiation alone to test for bystander effect (3A/3B vs expected survival): MDA-MB-231 cells were irradiated, allowed to repair for 1 day, and compared to the expected total reproductive cell survival based on the total irradiated area. That is, we assumed no clonogenic cell survival in the irradiated regions and full clonogenic survival in the lead-shielded regions.
5. Effect of repopulation (5A/5B vs. 3A/3B, respectively): Irradiated MDA-MB-231 cells were allowed to repopulate for 5 days following a 24 h period of post-irradiation repair and compared to the groups that allowed for post-irradiation repair only.
6. Local effect of radiation + PBMCs vs. radiation alone (4A/4B vs. 5A/5B, respectively): MDA-MB-231 cells were irradiated, allowed to repair for 1 day, and then either co-cultured with PBMCs (1:1 ratio) or not for 5 days.

### 4.2. Construction of GRID Collimators

To isolate the bystander effect, we constructed collimator grids from a 2 mm thick lead sheet. We used an Eastwood 14-gauge metal hand punch with a 13-gauge die (The Eastwood Company, Inc., Pottstown, PA, USA) to perforate the sheet with two-hole patterns designed to attenuate the primary beam, shielding different percentages of the cell population as follows:

1. Three 13-gauge holes (2.38 mm diameter) with 5 mm center-to-center spacing (Figure 1A), allowing for 12.8% of the cross-sectional area to receive 20 Gy.
2. Six 13-gauge holes with 4 mm center-to-center spacing (Figure 1B), allowing 25.7% of the cross-sectional area to receive 20 Gy.

These configurations minimized the radiation exposure to shielded cells, allowing us to evaluate the reproductive viability associated with the bystander effect.

### 4.3. Irradiator Set-Up and Dose Delivery Time

We used the CellRad X-ray irradiator (Precision X-ray, Inc., Madison, CT, USA) with turntable rotation enabled to irradiate the 3D cell culture scaffolds to a uniform dose. We achieved a 2 mm Al half-value layer beam quality using the 100 kV setting with a 0.5 mm Al filter added. Additionally, we verified the CellRad’s dose calibration in accordance with the AAPM TG-61 protocol [42].

**Figure 7:**
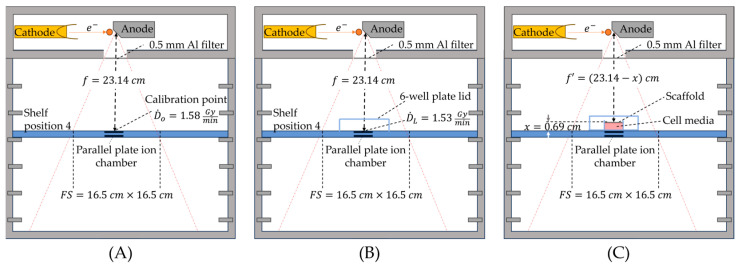
Irradiator set-up for dose delivery. (**A**) Calibrated dose rate (${\dot{D}}_{O}=1.58\;Gy/\min$) measured at the ionization chamber surface. (**B**) Dose rate (${\dot{D}}_{L}=1.53\;Gy/\min$) measured with the well plate lid in place. (**C**) Illustration of the distance used to calculate the dose delivery time to the cell culture scaffold using Equation (1). Note: The orange dot, red square, and double line depict an electron, cell media with 3D scaffold, and parallel plate ion chamber, respectively.

Figure 7 shows our dosimetric set-up to find the beam-on time for irradiating the Alvetex^®^ cell-cultured scaffolds with a 20 Gy peak dose. To account for the attenuation of our beam through the 24-well plate top lid (Corning, 3526, Glendale, AZ, USA), we measured the dose rate with and without the lid (${\dot{D}}_{L}=1.53\;Gy/min\;$ and ${\dot{D}}_{O}=1.58\;Gy/min,\;respectively)$ using the calibrated parallel-plate ionization chamber of the CellRad irradiator (Figure 7A,B). We also applied the inverse square law for the Alvetex^®^ surface distance x above the calibration dose point (Figure 7C). The final beam-on time $T_{L}$ required to deliver 20 Gy with the lid was computed as follows:

(1) $$T_{L}=T_{O}\cdot \frac{{\dot{D}}_{O}}{{\dot{D}}_{L}}\cdot {\left(\frac{f^{\prime }}{f}\right)}^{2}$$

where $T_{O}=12.66\;min$ is the beam-on time required to deliver 20 Gy without the lid, f=23.14 cm is the source-to-calibration point distance, and $f^{\prime }=f-x=23.14-0.69\;cm=22.45\;cm$ is the source-to-scaffold distance (Figure 7C). Hence, using Equation (1), 20 Gy was delivered to the scaffolds using a time of $T_{L}=12.30\;min$.

### 4.4. GRID Film Dosimetry Verification

We placed a Gafchromic^TM^ EBT3 film (Ashland Inc., Wilmington, DE, USA, Lot #06142101) on the CellRad’s motorized turntable to assess the beam flatness. The film’s calibration curve was generated in an open field without the presence of a lead grid or 24-well plate by irradiating triplicate film strips in 2 Gy increments from 0 to 6 Gy. We scanned all the films 1 day after exposure using an Epson Expression 10000XL flat-bottomed scanner (Epson America, Inc., Los Alamitos, CA, USA) at a 48-bit color depth and 72 dpi [43]. We generated a calibration curve for the red channel based on the ImageJ (Version 1.54i 03) protocol outlined by Howard et al. [44].

We used the CellRad ionization chamber to measure the dose rate ${\dot{D}}_{P}$ at the calibration point with the 24-well plate above the chamber and computed the time to deliver 5 Gy, using Equation (1), but replacing ${\dot{D}}_{L}$ with ${\dot{D}}_{P}$ to account for beam attenuation through the well plate. Next, we placed the lead grids on top of the well plate with the film below the well plate, at the same location as our calibration films, to obtain the peak-to-valley dose data shown in Figure 1B,D.

### 4.5. Culturing the MDA-MB-231 Cells

We cultured MDA-MB-231 cells from the American Type Culture Collection in T-25 Corning flasks using RPMI 1640 medium supplemented with L-glutamine additives (Gibco^TM^, Thermo Fisher Scientific, Inc., Waltham, MA, USA), 10% fetal bovine serum (FBS), and 1% penicillin–streptomycin (Gibco^TM^, Thermo Fisher Scientific, Inc.). We incubated the cells at 37 °C and 5% CO_2_ in a humidified Galaxy 170S incubator (New Brunswick, Inc., Moncton, NB, Canada).

We confirmed the absence of mycoplasma contamination using MycoStrip^TM^ (Invivogen, San Diego, CA, USA), and extracted cells from the T-25 flask using 0.25% trypsin (Gibco^TM^, Thermo Fisher Scientific, Inc.), followed by centrifugation (Cole-Palmer, Inc., Vernon Hills, IL, USA) at 195× *g* for 5 min. Post-centrifugation, we removed the cell pellet and resuspended it in approximately 1 mL of media. The cell concentration was found using the trypan blue exclusion assay with a Bright-Line^®^ hemocytometer (Hausser Scientific, Inc., Horsham, PA, USA), and the cells were diluted to $3.33\times 10^{5}$ cells/mL.

### 4.6. Culturing the MDA-MB-231 Cells in the 3D Scaffolds

We passaged the cells at least four times before seeding them onto Alvetex^®^ 3D cell culture scaffolds. The scaffolds were composed of highly porous polystyrene and provided a biomimetic cellular environment that promoted cell–cell interactions and maintained the natural 3D morphology and function of cells [29]. Each scaffold sat on a 12-well plate insert and had a diameter of 11.5 mm, a thickness of 200 µm, and 36–40 µm pores throughout to facilitate the maintenance of the natural cell shape and allow cancer cells to infiltrate the pores, forming small spheroids.

We first rehydrated the scaffolds with 4 mL of 70% ethanol for 10 min. We then aspirated the ethanol and washed the scaffolds twice with 4 mL of prepared RPMI 1640 medium, carefully avoiding excessive pipette force when washing or rehydrating the substrate to prevent scaffold damage. Following the second wash, we aspirated the medium and pipetted 75 µL of the $3.33\times 10^{5}$ cells/mL cell suspension onto the scaffolds. As per manufacturer specifications, we allowed the cells to settle into the scaffold for 15 min in a 37 °C incubator. To minimize cell detachment and agitation, we gently poured the prepared medium (4 mL per scaffold) onto the side of the insert rather than directly onto the cells. We then incubated the MDA-MB-231 culture scaffolds for 5 days at 37 °C and 5% CO_2_. After 5 days, approximately four cell-doubling times for this cell line, the scaffolds were prepared according to the groups shown in Table 1.

### 4.7. Irradiating the 3D Scaffolds

We transferred the scaffolds from the 12-well plate to a 24-well plate using sterile tweezers and added 1 mL of media to each well. The plate lid was placed on top and the lead grid was secured to the lid using four adhesive tabs. Following irradiation, we transferred the samples, their irradiated media, and 3 mL of fresh media back to 12-well plates for further processing.

### 4.8. Preparing the PBMCs for Co-Culture

We thawed normal human PBMCs procured from HumanCells Biosciences, Inc., according to the protocol described by Barcelo et al. [45]. We omitted the DNAse treatment, de-clumped the cells, and mixed them by the gentle flicking and racking of the tube.

Following thawing and mixing, we found the concentration of the PBMCs, diluted it to $1.6\times 10^{6}$ cells/mL using the prepared RPMI culture medium, and stored the cells in a conical tube.

### 4.9. Extracting MDA-MB-231 Cells from the Scaffold

To extract only the MDA-MB-231 cells from the scaffold, we used the Alvetex protocol (available online: https://www.reprocell.com/protocols/alvetex-scaffold/d054-cell-retrieval (accessed on 23 February 2024)), with the following modifications: We submerged each scaffold into a 50 mL conical tube, added 4 mL of 0.25% trypsin EDTA, and incubated it for 15 min. After incubation, we gently mixed and flicked the tube by hand to dislodge cells from the scaffold and washed the trypsin through the scaffold to maximize recovery. Finally, we pipetted the mixture into a new conical tube and centrifuged it at 195× *g* for 5 min.

### 4.10. Clonogenic Assay

We followed a previously published protocol for the clonogenic assay [46]. We seeded 2 mL of cell-containing media into each well of pre-treated 6-well plates (Nest Scientific, Inc., Woodbridge, NJ, USA), providing six technical replicates and at least three biological replicates per experimental group. We incubated the seeded MDA-MB-231 cells for 10 days to promote colony growth and replaced the media on day five of incubation. On day 10, we aspirated the media and fixed, stained, and took images of the wells. We used the images to manually count colonies (at least 50 cells) using FiJi’s cell counter plugin (Version 2.16.0) [47]. We calculated the plating efficiency for the control samples (Equation (2)) and normalized the colony counts to the controls (Equation (3)) to compute the surviving fractions and assess the effectiveness of the tested parameters on the reproductive viability of the MDA-MB-231 cells:

(2) $$\mathrm{Plating}\;\mathrm{Efficiency}=\frac{Number\;of\;colonies\;counted}{Number\;of\;cells\;seeded}$$

(3) $$\mathrm{Surviving}\;\mathrm{Fraction}=\frac{Number\;of\;colonies\;counted}{Number\;of\;cells\;seeded*Plating\;Efficiency}$$

### 4.11. Local Effect of PBMCs Alone on MDA-MB-231 Cells

After incubating the cancer cells within the scaffold for 5 days, we aspirated and replaced the media with 3.75 mL of supplemented RPMI 1640. We added 250 µL of PBMCs to the cancer cell scaffold, achieving a 1:1 ratio of MDA-MB-231 cancer cells to PBMCs. We incubated this co-culture at 37 °C with 5% CO_2_ for 5 days. On the fifth day, the scaffold was removed from the co-culture and the cells were extracted. We counted the extracted MDA-MB-231 cells using the trypan blue exclusion assay and plated them for the clonogenic assay.

### 4.12. Simulating the Abscopal Effect

To model the abscopal effect, samples were irradiated in triplicate using 3- or 6-hole lead grids. Concurrently, we cultured non-irradiated MDA-MB-231 scaffolds to represent distant metastases. After 1 day, the irradiated and non-irradiated 3D culture scaffolds were swapped using sterile tweezers, and PBMCs were added to create a 1:1 co-culture. In this experiment, the irradiated samples mimicked the primary tumor receiving GRID-RT, while the non-irradiated samples, now exposed to ICM, represented distant metastases. After 5 days, the MDA-MB-231 cells were extracted and counted to assess if the ICM exposure of the non-irradiated samples primed with PBMCs induced an abscopal effect.

### 4.13. Expected Surviving Fraction from GRID Irradiation

The scaffolds, which had a 103.87 mm^2^ cross-sectional growth area, were subjected to 3- and 6-hole GRID irradiation, resulting in 12.8% and 25.7% of the total scaffold cross-sectional area being irradiated, respectively. To err on the side of overestimating cell death in irradiated regions, we assumed complete cell ablation in the 20 Gy peak-dose irradiation regions. This deliberate conservative assumption yielded the expected lower bounds for cell survival, namely, 100 − 12.8% = 87.2% and 100 − 25.7% = 74.3% for the 3- and 6-hole GRIDs, respectively. This implies that an observed cell kill of even less than these lower limits was the result of the bystander effect. Hence, these expected lower bounds served as a baseline for comparing the actual impact of GRID irradiation on reproductive cell survival within our scaffold system to quantify the minimal RIBE.

### 4.14. Assessing the RIBE with GRID Irradiation

We investigated the RIBE due to a high-dose GRID treatment by first irradiating the scaffolds with a 20 Gy peak dose using the 3- and 6-hole GRIDs, with average hole separations of 2.6 mm and 1.6 mm, respectively (Figure 1A,C). Following irradiation, we incubated the inserts for 1 day to allow for DNA repair, extracted the cells, and plated them for the clonogenic survival assay. To evaluate the RIBE, we compared the experimentally observed reproductive cell survivals with the expected cell survivals described in Section 4.13.

### 4.15. Combined RIBE and Immune-Mediated Response

To assess the combined effects of the high-dose RIBE and PBMC-mediated immune responses on cancer cell viability, we irradiated the MDA-MB-231 cells according to their designated group (Table 1). Following irradiation, we allowed a 24 h repair period. We then introduced PBMCs into the culture in a 1:1 ratio and maintained this co-culture for 5 days. After the co-culture period, we removed the 3D scaffolds, extracted the MDA-MB-231 cells, counted them, and seeded them for the clonogenic assay.

### 4.16. GRID Effect on Irradiated and Non-Irradiated Clonogenic Survival

To evaluate the impact of GRID irradiation on reproductive cell viability in co-culture, we first irradiated the samples according to the groups designated in Table 1 and allowed a 24 h repair period followed by repopulation for 5 days. After this period, we extracted the cells from the 3D scaffolds and seeded the cells for the clonogenic assay. In parallel, we irradiated a separate set of scaffolds, allowed for 1 day for repair, and then we swapped them with non-irradiated scaffolds to assess the influence of ICM post-irradiated cell contents on viability. Following the swap, we allowed the cells to repopulate for 5 days, then extracted and seeded them for the clonogenic assay.

### 4.17. Statistical Analysis

We formulated directional hypotheses for the group comparisons, anticipating a decrease in the mean reproductive cell survival with increasing treatment intensity. We expected the combined radiation and PBMC group to exhibit the lowest survival. The normality of the data and the homogeneity of the variance were assessed using the Shapiro–Wilk and Levene’s tests, respectively. As all the groups met these assumptions, we conducted a one-way ANOVA for multiple group comparisons (C2, 1A, and 1B vs. C1) and one-tailed *t*-tests for pairwise comparisons. Statistical analyses were performed in Excel using the Real Statistics add-in, with a significance level of α = 0.05.

## 5. Conclusions

Despite the aforementioned limitations, our study provides valuable insights into the bystander and abscopal effects of GRID irradiation in a 3D co-culture scaffold model. Our findings suggest that the high-dose RIBE plays a significant role in the therapeutic efficacy of GRID-RT, while the abscopal effect requires further optimization to be clinically relevant. Our results also indicate an “overkill” effect in the overlapping bystander regions between the holes, which has important implications for the optimal design of grid-hole spacings. Lastly, the 3D scaffold model presented in this study offers a promising platform for future investigations into the mechanisms and therapeutic potential of GRID irradiation, alone and in combination with immunotherapies.

## Figures and Tables

**Table 1:** Experimental groups and conditions. ICM = irradiated conditioned media (i.e., the media transferred from 3D MDA-MB-231 culture scaffolds irradiated without PBMCs); R & R = repair and repopulation; Primary = directly irradiated cells in the 3D scaffolds. The percent cell survival column gives the mean ± standard deviation.

Group	Radiation Peak Dose (Gy)	PBMCs	ICM	Post-Treatment Incubation Time (Days)	% Cell Survival
C1. Control	0	No	No	NA	(100 ± 18)%
C2. PBMC Control	0	Yes	No	5	(72 ± 5)%
1A. ICM: 3-Hole GRID	0	No	Yes	5	(105 ± 16)%
1B. ICM: 6-Hole GRID	0	No	Yes	5	(78 ± 13)%
2A. Abscopal: 3-Hole GRID	0	Yes	Yes	5	(74 ± 5)%
2B. Abscopal: 6-Hole GRID	0	Yes	Yes	5	(79 ± 12)%
3A. Bystander: 3-Hole GRID	20	No	Yes	1	(68 ± 4)%
3B. Bystander: 6-Hole GRID	20	No	Yes	1	(54 ± 14)%
4A. Primary: 3-Hole GRID	20	Yes	Yes	6	(61 ± 6)%
4B. Primary: 6-Hole GRID	20	Yes	Yes	6	(59 ± 7)%
5A. R & R: 3-Hole GRID	20	No	Yes	6	(91 ± 6)%
5B. R & R: 6-Hole GRID	20	No	Yes	6	(86 ± 22)%

## Data Availability

Please contact the corresponding author (M.T.) for the raw data.
